# Supplementary material for: Genetics of Plasma Bilirubin and Associations between Bilirubin and Cardiometabolic Risk Profiles in Danish Children and Adolescents
Source: Antioxidants (Basel). 2023 Aug 15;12(8):1613. doi: 10.3390/antiox12081613 (PMC10451688; doi:10.3390/antiox12081613)
Supplement: Supplementary file 1 [file antioxidants-12-01613-s001.zip › antioxidants-2483945-supplementary.pdf]

Supplementary Materials

Table S1. Estimated regression β-effects (95% CI) for associations of plasma bilirubin with cardiometabolic risk factors in a pooled model.

Cardiometabolic Risk Factor	Predictor	Model	beta_CI	*p*-Value
ALT	Bilirubin	1	0.02 (0.01, 0.03)	0.00024
ALT	Bilirubin	2	0.01 (0, 0.02)	0.005
hs-CRP	Bilirubin	1	−0.01 (−0.02, 0)	0.0088
hs-CRP	Bilirubin	2	−0.01 (−0.02, 0)	0.0027
HOMA-IR	Bilirubin	1	−0.04 (−0.05, −0.03)	1.92×10^{-15}
HOMA-IR	Bilirubin	2	−0.04 (−0.04, −0.03)	1.6×10^{-12}
HbA1c	Bilirubin	1	−0.04 (−0.05, −0.03)	5.54×10^{-18}
HbA1c	Bilirubin	2	−0.04 (−0.05, −0.03)	9.29×10^{-16}
HDL-C	Bilirubin	1	0.01 (0, 0.02)	0.04
HDL-C	Bilirubin	2	0.01 (0, 0.02)	0.08
LDL-C	Bilirubin	1	−0.01 (−0.02, 0)	0.07
LDL-C	Bilirubin	2	−0.01 (−0.02, 0)	0.030
Triglycerides	Bilirubin	1	−0.02 (−0.03, −0.01)	4.8×10^{-5}
Triglycerides	Bilirubin	2	−0.02 (−0.03, −0.01)	0.0003
SBP SDS	Bilirubin	1	0.01 (0, 0.02)	0.020
SBP SDS	Bilirubin	2	0.01 (0, 0.02)	0.09
DBP SDS	Bilirubin	1	−0.01 (−0.01, 0)	0.26
DBP SDS	Bilirubin	2	−0.01 (−0.02, 0)	0.13
BMI SDS*	Bilirubin	1	−0.01 (−0.02, 0)	0.012
BMI SDS*	Bilirubin	2	−0.01 (−0.02, 0)	0.05
Waist SDS*	Bilirubin	1	−0.01 (−0.02, 0)	0.012
Waist SDS*	Bilirubin	2	−0.01 (−0.02, 0)	0.026
Body fat %*	Bilirubin	1	−0.01 (−0.02, 0.01)	0.23
Body fat %*	Bilirubin	2	−0.02 (−0.03, 0)	0.02

Model 1: cardiometabolic risk factor (SD units) ~ bilirubin + age + sex + smoking status (passive smoking, non-smoking) + BMI SDS.
Model 2: cardiometabolic risk factor (SD units) ~ bilirubin + age + sex + smoking status (passive smoking, non-smoking) + BMI SDS + puberty stage.
Abbreviations: *, represents cardiometabolic risk factors, which were not adjusted for BMI SDS. ALT, alanine aminotransferase; CI, confidence interval; DBP, diastolic blood pressure; HbA1c, hemoglobin A1c; HDL-C, high-density lipoprotein cholesterol; HOMA-IR, homeostasis model assessment of insulin resistance; hs-CRP, high-sensitivity C-reactive protein; LDL-C, low-density lipoprotein cholesterol; SBP, systolic blood pressure; SDS, standard deviation score.

Table S2. Estimated regression β-effects (95% CI) for associations of bilirubin and genotype of SNP rs887829 with plasma inflammatory markers in a pooled model.

Marker	Predictor	beta_CI	*p*-value	Predictor	beta_CI	*p*-Value
VEGFA	Bilirubin	−0.13 (−0.17, −0.09)	2.56×10^{-9}	rs887829	−0.02 (−0.06,0.03)	0.5
OPG	Bilirubin	−0.13 (−0.17, −0.08)	4.86×10^{-9}	rs887829	0.009 (−0.04,0.055)	0.7
CCL20	Bilirubin	−0.13 (−0.17, −0.09)	3.77×10^{-9}	rs887829	−0.000 (−0.05,0.05)	0.99
IDUA	Bilirubin	−0.12 (−0.16, −0.07)	1.77×10^{-7}	rs887829	−0.019 (−0.067, 0.028)	0.43
CTRC	Bilirubin	−0.12 (−0.16, −0.08)	6.97×10^{-8}	rs887829	−0.05 (−0.099, −0.0039)	0.05
CCL11	Bilirubin	−0.11 (−0.16, −0.07)	5.02×10^{-7}	rs887829	0.041 (−0.007, 0.09)	0.09
LEP	Bilirubin	−0.11 (−0.14, −0.09)	2.42×10^{-21}	rs887829	−0.02 (−0.04, 0.009)	0.22
LPL	Bilirubin	−0.1 (−0.15, −0.06)	1.1×10^{-6}	rs887829	−0.035 (−0.08, 0.011)	0.13
FABP2	Bilirubin	−0.1 (−0.14, −0.06)	5.48×10^{-6}	rs887829	0.01 (−0.03, 0.06)	0.60
MARCO	Bilirubin	−0.1 (−0.15, −0.06)	1.02×10^{-6}	rs887829	−0.027 (−0.072, 0.019)	0.3

CDCP1	Bilirubin	−0.09 (−0.13, −0.05)	5.89×10^{-6}	rs887829	0.014 (−0.03, 0.06)	0.5
IL18R1	Bilirubin	−0.09 (−0.13, −0.05)	2.25×10^{-5}	rs887829	−0.008 (−0.05, 0.04)	0.7
AMBP	Bilirubin	−0.09 (−0.13, −0.05)	2.17×10^{-5}	rs887829	−0.04 (−0.09, 0.003)	0.07
CCL19	Bilirubin	−0.08 (−0.12, −0.03)	0.0005	rs887829	0.006 (−0.041, 0.053)	0.8
CXCL10	Bilirubin	−0.08 (−0.12, −0.03)	0.00058	rs887829	0.006 (−0.041, 0.053)	0.8
MCP2	Bilirubin	−0.08 (−0.13, −0.04)	0.00016	rs887829	−0.0084 (−0.056, 0.39)	0.73
CCL25	Bilirubin	−0.08 (−0.12, −0.03)	0.00042	rs887829	0.04 (−0.007, 0.09)	0.09
SERPINA12	Bilirubin	−0.08 (−0.12, −0.03)	0.00040	rs887829	−0.02 (−0.06, 0.03)	0.42
IL7	Bilirubin	−0.07 (−0.11, −0.02)	0.0021	rs887829	0.0001 (−0.05, 0.05)	0.99
CXCL11	Bilirubin	−0.07 (−0.12, −0.03)	0.0005	rs887829	0.016 (−0.031, 0.06)	0.51
MMP1	Bilirubin	−0.07 (−0.11, −0.02)	0.0028	rs887829	0.0069 (−0.039, 0.053	0.8
PDL1	Bilirubin	−0.07 (−0.11, −0.03)	0.0012	rs887829	0.016 (−0.03, 0.063)	0.5
MMP10	Bilirubin	−0.07 (−0.12, −0.03)	0.00094	rs887829	0.044 (−0.003, 0.09)	0.06
IFNg	Bilirubin	−0.07 (−0.12, −0.03)	0.00096	rs887829	−0.004 (−0.052, 0.044)	0.87
FGF19	Bilirubin	−0.07 (−0.11, −0.03)	0.0018	rs887829	−0.0096 (−0.057, 0.038)	0.69
MMP12	Bilirubin	−0.07 (−0.11, −0.03)	0.0003	rs887829	−0.004 (−0.05, 0.04)	0.9
AXIN1	Bilirubin	−0.06 (−0.1, −0.02)	0.006	rs887829	−0.02 (−0.064, 0.028)	0.45
CXCL9	Bilirubin	−0.06 (−0.1, −0.02)	0.0075	rs887829	0.031 (−0.02, 0.08)	0.2
SLAMF1	Bilirubin	−0.06 (−0.1, −0.02)	0.0076	rs887829	0.022 (−0.025, 0.069)	0.4
SIRT2	Bilirubin	−0.06 (−0.11, −0.02)	0.0033	rs887829	−0.0003 (−0.47, 0.047)	0.99
CCL28	Bilirubin	−0.06 (−0.1, −0.02)	0.0036	rs887829	0.019 (0.026, 0.07)	0.4
STAMBP	Bilirubin	−0.06 (−0.1, −0.02)	0.0058	rs887829	−0.004 (−0.051, 0.042)	0.9
TNFRSF10A	Bilirubin	−0.06 (−0.1, −0.02)	0.0075	rs887829	−0.009 (−0.06, 0.04)	0.7
STK4	Bilirubin	−0.06 (−0.11, −0.02)	0.0046	rs887829	−0.02 (−0.07, 0.027)	0.4
PRSS8	Bilirubin	−0.06 (−0.1, −0.02)	0.005	rs887829	0.014 (−0.032, 0.06)	0.6
IL8	Bilirubin	−0.05 (−0.1, −0.01)	0.0161	rs887829	0.0021 (−0.045, 0.05)	0.9
LAP_TGFb1	Bilirubin	−0.05 (−0.1, −0.01)	0.0131	rs887829	0.009 (−0.04, 0.057)	0.7
IL17C	Bilirubin	−0.05 (−0.1, −0.01)	0.0129	rs887829	−0.044 (−0.09, 0.003)	0.07
CD40L	Bilirubin	−0.05 (−0.1, −0.01)	0.014	rs887829	0.002 (−0.05, 0.05)	0.9
IL1ra	Bilirubin	−0.05 (−0.09, −0.02)	0.001	rs887829	−0.007 (−0.043, 0.03)	0.7
SORT1	Bilirubin	−0.05 (−0.1, −0.01)	0.0121	rs887829	0.006 (0.04, 0.05)	0.8
GH	Bilirubin	0.05 (0.01, 0.09)	0.0161	rs887829	0.020 (−0.022, 0.06)	0.4
PTX3	Bilirubin	0.05 (0.01, 0.09)	0.008	rs887829	−0.005 (−0.047, 0.036)	0.8
THBS2	Bilirubin	0.06 (0.01, 0.1)	0.014	rs887829	0.021 (−0.027, 0.069)	0.4
TNFRSF9	Bilirubin	0.07 (0.03, 0.1)	0.00032	rs887829	0.015 (−0.024, 0.05)	0.4
PGF	Bilirubin	0.07 (0.03, 0.11)	0.0019	rs887829	−0.002 (−0.05, 0.045)	0.9
PAPPA	Bilirubin	0.07 (0.03, 0.11)	0.0015	rs887829	−0.033 (−0.08, 0.014)	0.2
GT	Bilirubin	0.07 (0.03, 0.11)	0.0012	rs887829	−0.003 (−0.05, 0.044)	0.9
CCL23	Bilirubin	0.08 (0.04, 0.12)	0.0003	rs887829	0.012 (−0.04, 0.059)	0.6
CX3CL1	Bilirubin	0.09 (0.05, 0.13)	5.41×10^{-5}	rs887829	0.010 (−0.037, 0.058)	0.7
SCF	Bilirubin	0.1 (0.06, 0.15)	2.61×10^{-6}	rs887829	−0.009 (−0.056, 0.038)	0.7
TRANCE	Bilirubin	0.11 (0.07, 0.15)	1.55×10^{-7}	rs887829	0.033 (−0.012, 0.077)	0.2

Abbreviations: CCL20, C-C motif chemokine ligand 20; VEGF, vascular endothelial growth factor A; OPG, osteoprotegerin; CTRC, chymotrypsin C; IDUA, alpha-L-iduronidase; LEP, leptin; CCL11, C-C motif chemokine ligand 11; LPL, lipoprotein lipase; MARCO, macrophage receptor with collagenous structure; FABP2, fatty acid-binding protein 2; CDCP1, CUB domain-containing protein 1; IL18R1, interleukin 18 receptor 1; AMBP, alpha-1-microglobulin/bikunin precursor; MCP2, monocyte chemotactic protein 2; CCL25, C-C motif chemokine ligand 25; CXCL10, C-X-C motif chemokine ligand 10; SERPINA12, serpin family A member 12; CCL19, C-C motif chemokine ligand 19; CXCL11, C-X-C motif chemokine ligand 11; IFNg, interferon gamma; MMP10, matrix metallopeptidase 10; MMP12, matrix metallopeptidase 12; PDL1, programmed cell death 1 ligand 1; FGF19, fibroblast growth factor 19; IL7, interleukin 7; MMP1, matrix metallopeptidase 1; SIRT2, sirtuin 2; STK4, serine/threonine kinase 4; CCL28, C-C motif chemokine ligand 28; AXIN1, axin 1; STAMBP, STAM binding protein; SLAMF1, signaling lymphocytic activation molecule family member 1; PRSS8, serine

protease 8; CXCL9, C-X-C motif chemokine ligand 9; TNFRSF10A, TNF receptor superfamily member 10a; IL17C, interleukin 17C; SORT1, sortilin 1; LAP_TGFb1, latency-associated peptide transforming growth factor beta-1; IL1ra, interleukin-1 receptor antagonist; CD40L, cluster of differentiation 40 ligand; IL8, interleukin 8; GH, growth hormone; PTX3, pentraxin 3; THBS2, thrombospondin 2; TNFRSF9, TNF receptor superfamily member 9; PGF, placental growth factor; PAPPA, pappalysin 1; GT, gastrotropin; CCL23, C-C motif chemokine ligand 23; CX3CL1, C-X3-C motif chemokine ligand 1; SCF, stem cell factor; TRANCE, TNF-related activation-induced cytokine.

Table S3. Estimated regression β-effects (95% CI) for associations of SNP rs887829 with cardiometabolic risk factors in a pooled model.

Cardiometabolic Risk Factor	Predictor	Model	beta_CI	*p*-Value
ALT	rs887829	1	−0.01 (−0.06, 0.04)	0.685
ALT	rs887829	2	−0.01 (−0.06, 0.04)	0.720
hs-CRP	rs887829	1	0.06 (0.01, 0.1)	0.012
hs-CRP	rs887829	2	0.06 (0.01, 0.1)	0.011
HOMA-IR	rs887829	1	0.01 (−0.04, 0.06)	0.634
HOMA-IR	rs887829	2	0.01 (−0.04, 0.06)	0.621
HbA1c	rs887829	1	0.02 (−0.03, 0.07)	0.460
HbA1c	rs887829	2	0.02 (−0.03, 0.07)	0.470
HDL-C	rs887829	1	−0.04 (−0.08, 0.01)	0.098
HDL-C	rs887829	2	−0.04 (−0.09, 0.01)	0.096
LDL-C	rs887829	1	−0.02 (−0.07, 0.03)	0.362
LDL-C	rs887829	2	−0.02 (−0.07, 0.03)	0.365
Triglycerides	rs887829	1	0.03 (−0.02, 0.07)	0.263
Triglycerides	rs887829	2	0.03 (−0.02, 0.07)	0.257
Bilirubin	rs887829	1	0.76 (0.72, 0.81)	6.009×10^{-214}
Bilirubin	rs887829	2	0.76 (0.72, 0.81)	2.1167×10^{-213}
SBP SDS	rs887829	1	0.04 (−0.01, 0.09)	0.115
SBP SDS	rs887829	2	0.04 (−0.01, 0.09)	0.132
DBP SDS	rs887829	1	0 (−0.05, 0.05)	0.900
DBP SDS	rs887829	2	0 (−0.05, 0.05)	0.902
BMI SDS*	rs887829	1	0.04 (−0.01, 0.09)	0.098
BMI SDS*	rs887829	2	0.04 (−0.01, 0.09)	0.099
Waist SDS*	rs887829	1	0.03 (−0.02, 0.08)	0.184
Waist SDS*	rs887829	2	0.03 (−0.02, 0.08)	0.179
Body fat %*	rs887829	1	0.03 (−0.06, 0.12)	0.536
Body fat %*	rs887829	2	0.03 (−0.06, 0.12)	0.549

Model 1: cardiometabolic risk factor (SD units) ~ genotype + age + sex + smoking status (passive smoking, non-smoking) + BMI SDS.
Model 2: cardiometabolic risk factor (SD units) ~ genotype + age + sex + smoking status (passive smoking, non-smoking) + BMI SDS + puberty stage.
Abbreviations: *, represents cardiometabolic risk factors, which were not adjusted for BMI SDS. ALT, alanine aminotransferase; CI, confidence interval; DBP, diastolic blood pressure; HbA1c, hemoglobin A1c; HDL-C, high-density lipoprotein cholesterol; HOMA-IR, homeostasis model assessment of insulin resistance; hs-CRP, high sensitivity C-reactive protein; LDL-C, low-density lipoprotein cholesterol; SBP, systolic blood pressure; SDS, standard deviation score.

Table S4. The association between plasma bilirubin concentrations and cardiometabolic risk features.

Cardiometabolic Risk Feature	Model	OR (95% CI)	*p*-Value
Insulin resistance	1	0.69 (0.59, 0.79)	2.10×10^{-12}
Hyperglycemia	1	0.86 (0.74 , 0.88)	0.0096
Dyslipidemia	1	0.89 (0.8 , 0.95)	0.018
Hypertension	1	1.01 (0.90 , 1.05)	0.93
High ALT	1	1.02 (0.93 , 1.05)	0.64

Insulin resistance	2	0.67 (0.55 , 0.75)	6.90×10^{-12}
Hyperglycemia	2	0.82 (0.69 , 0.86)	0.0041
Dyslipidemia	2	0.91 (0.81 , 0.98)	0.04
Hypertension	2	0.99 (0.87 , 1.02)	0.84
High ALT	2	0.98 (0.89 , 1.03)	0.72

Table S5. The association between genotype of SNP rs887829 and cardiometabolic risk features.

Cardiometabolic Risk Feature	Model	OR (95% CI)	*p*-Value
Insulin resistance	1	1.04 (0.86 , 1.15)	0.61
Hyperglycemia	1	1.14 (0.98 , 1.26)	0.12
Dyslipidemia	1	0.97 (0.83 , 1.15)	0.69
Hypertension	1	1.09 (0.93 , 1.34)	0.31
High ALT	1	1 (0.87 , 1.17)	1.0
Insulin resistance	2	1.01 (0.85 , 0.14)	0.91
Hyperglycemia	2	1.14 (0.96 , 1.16)	0.15
Dyslipidemia	2	0.94 (0.79 , 1.14)	0.42
Hypertension	2	1.14 (0.96 , 1.20)	0.14
High ALT	2	0.97 (0.83 , 1.15)	0.68
